# Clinical Characteristics and Outcomes of Patients Managed with Percutaneous Dilatational Tracheostomy in the Intensive Care Unit: A Retrospective Observational Study

**DOI:** 10.31729/jnma.9066

**Published:** 2025-06-30

**Authors:** Utsav Lal Pradhan, Bipin Karki, Hem Raj Paneru, Pramesh Sunder Shrestha, Gentle Sunder Shrestha, Subhash Prasad Acharya

**Affiliations:** 1 Department of Critical Care Medicine, Tribhuvan University Teaching Hospital, Maharajgunj, Kathmandu, Nepal

**Keywords:** *critical care*, *ICU outcomes*, *mechanical ventilation*, *neurological disorders*, *percutaneous dilatational tracheostomy*

## Abstract

**Introduction:**

Percutaneous dilatational tracheostomy (PDT) has emerged as a preferred minimally invasive alternative to surgical tracheostomy for critically ill patients requiring prolonged mechanical ventilation, though comprehensive data from Nepal remains limited. This study aimed to evaluate the practice of PDT and analyze clinical outcomes in a tertiary level ICU in Nepal.

**Methods:**

A retrospective observational study was conducted at Level III ICUs Nepal from April 14, 2021 to April 12, 2024. All patients aged 18 and older who underwent percutaneous dilatational tracheostomy during ICU stay in the study duration were included. Ethical approval for the study was obtained from the institutional review committee [Ref. 583. (6-11) E2]. Data on clinical characteristics, procedural techniques, complications, and outcomes were collected from electronic records and individual file records from hospital record department.

**Results:**

The study population comprised predominantly males 53 (63.86%) with a median age of 49 (IQR: 30-62) years. Neurological disorders, particularly intracranial haemorrhage, were the most common admission diagnosis 53 (63.86%). The mean duration from mechanical ventilation to PDT was 16 (IQR: 11-20) days and the main indication was prolonged mechanical ventilation 62 (74.7%). Grigg's guidewire dilator forceps technique was used 77 (92.77%) of the time. Immediate complications occurred in 12 (14.46%) of cases. The hospital survival rate was 42 (50.6%) with 69 % of survivors achieving decannulation before discharge.

**Conclusions:**

PDT is a safe bedside procedure for critically ill patients needing prolonged ventilation, with only minor, non-life-threatening complications observed in our tertiary care setting.

## INTRODUCTION

Critically ill patients requiring prolonged mechanical ventilation frequently require a long-term airway, with studies showing up to 8 -30% of mechanically ventilated patients in ICUs ultimately needing tracheostomy.^1-2^ Percutaneous Dilatational Tracheostomy (PDT) has emerged as the preferred minimally invasive alternative to traditional surgical tracheostomy in ICUs worldwide due to its relative ease of performance, shorter procedural time and lower complication rates. ^3-6^

The patient population undergoing PDT exhibit diverse medical conditions, comorbidities and varying illness severity.^7-9^ Despite PDT's increasing utilization, comprehensive data on patient profiles, outcomes and complications remain limited, particularly in Nepal's healthcare context. Recent studies in Nepal have highlighted inconsistent PDT practices with small sample size for validation of outcomes and complications.^10-12^

This study aimed to identify the clinical profiles of these patients undergoing PDT including clinical demographics, in ICU complications and outcomes including length of ICU stay, hospital stay and decannulation before discharge.

## METHODS

This was a retrospective observational study that was conducted at the Department of Critical Care Medicine at Tribhuvan University Teaching Hospital (TUTH), Nepal in two level III ICUs from April 14, 2021 (Baisakh 1, 2078) to April 12, 2024 (Chaitra 30, 2080). TUTH is an 850 bedded center for patients from all across the country. The ICUs at TUTH is an intensivist led ICU comprising 22 level III ICU beds in two blocks (ICU A and ICU B) providing services to medical and surgical patients.

Ethical approval for the study was obtained from the institutional review committee dated May 15, 2024 [Ref. 583. (6-11) E2] Institutional Review Committee granted a waiver of consent due to retrospective nature of the study.

We included all patients aged 18 and older who underwent percutaneous dilatational tracheostomy during ICU stay in a 3-year period from April 14, 2021 to April 12, 2024. Patients with incomplete medical records were excluded.

Patient data were extracted from electronic health records and individual patient file records and compiled into a comprehensive data collection sheet. The variables collected included age, gender, APACHE II score, body mass index (BMI), admission type (medical/surgical), admission diagnosis, comorbidities, procedural characteristics including days of mechanical ventilation prior to PDT, indication and technique of PDT, immediate complications of PDT, and clinical outcome measures including length of mechanical ventilation, ICU stay, hospital stay, decannulation and survival recorded. Data was expressed as mean ± standard deviation for normally distributed variables and median with interquartile range (IQR) for non-normally distributed variables. Categorical variables were presented as frequencies and percentages. All data analysis were conducted using Microsoft Excel Version 16.78.

## RESULTS

Of the 3326 patients admitted during the three-year period. There were a total of 151 tracheostomies performed. Among them, 99 patients had undergone percutaneous tracheostomy and 16 patients were excluded due to missing records and 83 patients were included in the final analyis.

There were 53 (63.86%) males and 30 (36.14%) females, with a median age of 49 years (IQR: 30-62). The mean body mass index (BMI) was 24.67±3.34 kg/m^2^ and the mean APACHE II score was 20.19±7.33. Neurological disorders were the predominant admission diagnosis (n=53, 63.86%) with intracranial haemorrhage being the most frequent (n = 17, 20.48%), followed by traumatic brain injury (n = 8,9.64%), CNS infections (n = 6, 7.23%) and subarachnoid hemorrhage (n = 6, 7.23%). There were 39 (46.9%) patients with various comorbidities with systemic hypertension (n = 25, 39.29%) and chronic obstructive pulmonary disease (n = 12, 14.46%) being the most common comorbidities (Table 1).

**Table 1:** Baseline characteristics, Admission Diagnosis and Comorbidities of Patients with Percutaneous Dilational Tracheostomy(PDT) (n=83).

Baseline Characteristics of Patients with PDT		n(%)
Age: Median (Q1-Q3)		49(30-62)
Gender (N%)	Male	53(63.86%)
	Female	30(36.14%)
Admission type (N%)	Medical	34(40.96)
	Surgical	49(59.04)
**Body Mass Index: (Mean±SD)**		24.67±3.34
APACHE II (Mean±SD)		20.19±7.33
Admission Diagnosis		n(%)
Neurological		53(63.86)
Intracranial Haemorrhage (ICH)		17(20.48)
Traumatic Brain Injury		8(9.64)
CNS infection		6(7.23)
Subarachnoid Haemorrhage (SAH)		6(7.23)
Neuromuscular disease		5(6.02)
Brain Tumour		6(7.23)
Acute Ischemic Stroke (AIS)		3(3.61)
Status epilepticus		2(2.41)
Respiratory		9(10.84)
Pneumonia/Acute Respiratory Distress		9(10.84)
Syndrome (ARDS)	
Cardiac		3(3.61)
Heart Failure		3(3.61)
Others		18(21.69)
Trauma		7(8.43)
Post CPR		4(4.82)
Pancreatitis		2(2.41)
Chronic Kidney Disease (CKD)		1(1.2)
GI malignancy		1(1.2)
Poisoning		1(1.2)
Chronic Liver Disease		1(1.2)
Autoimmune Disorder		1(1.2)
Comorbidities		
Systemic Hypertension		25(39.29)
Chronic Obstructive Pulmonary Disease (COPD)		12(14.46)
Hypothyroidism		6(7.23)
Malignancy		5 (6.02)
Chronic Kidney Disease (CKD)		5 (6.02)
Heart failure		4 (4.82)
Peripheral Vascular disease		3 (3.61)
Stroke		2 (2.41)
Liver disease		2 (2.41)
Acute Coronary Syndrome		2 (2.41)
Valvular Heart Disease		1 (1.2)
Rheumatoid Arthritis		1 (1.2)

The median time from mechanical ventilation initiation to PDT was 16 days (IQR: 11-20). The primary indication of PDT was prolonged mechanical ventilation (n =62, 74.7%) followed by inability to protect airway (n=21, 25.3%). Grigg's technique was predominantly used in 77 (92.77%) cases with Ciaglia's Blue Rhino Serial Dilator technique utilized in 6 (7.23%) cases. The survival rate was 50.6% (42 patients), with 69% (29 patients) achieving successful decannulation before discharge. Among survivors, the median mechanical ventilation duration was 28.5 days (IQR: 20-41.5), median ICU length of stay was 32 days (IQR: 22.3-44.8), and median hospital length of stay was 45.5 days (IQR: 35-67) (Table 2).

**Table 2:** Procedural Characteristics and Clinical Outcomes of PDT (n=83).

Procedural Characteristics		n(%)
Mechanical Ventilation Days Prior to PDT: Median (Q1-Q3)		16(11-20)
Indication of Tracheostomy (N%)	Prolonged MV	62(74.7%)
	Inability to protect airway	21(25.3%)
Technique of Tracheostomy (N%)	Grigg's Guide Wire Dilator Forceps Technique	77(92.77%)
	Ciaglia's Blue Rhino Serial Dilator Technique	6(7.23%)
Clinical Outcomes		
Duration of Mechanical ventilation (Days) Median (Q1-Q3)		28.5(20-41.5)
ICU length off stay (Days) Median (Q1-Q3)		32(22.3-44.8)
Hospital lenght of stay (Days) Median (Q1-Q3)		45.5(35-67)
**Decannulation**		
Yes		29(34.94%)
No		54(65.06%)
Final Outcome		
Survivor		42(50.6%)
Non-Survivor		41(49.4%)

Immediate complications occurred in 12 (14.46%) patients, including minor bleeding in 9 (10.84%), transient hypotension in 2 (2.41%) and subcutaneous emphysema in 1 (1.2%) cases (Table 3).

**Table 3:** Complications of PDT (n=83).

Complications	n(%)
Minor Bleeding	9(10.84%)
Transient hypotension	2(2.41%)
Subcutaneous emphysema	1(1.20%)
Total	12(14.46%)

## DISCUSSION

This study showed that the majority of percutaneous dilatational tracheostomies were done for patients with neurological conditions (n = 53, 63.86%). PDTs were done at a median duration of 16 days following initiation of mechanical ventilation. The primary indication of PDTs were for prolonged mechanical ventilation (n = 62, 74.7%). TThe predominant technique of PDT was Grigg's guidewire dilator forceps technique used in 77 (92.7%) of the cases. Twelve patients (14.46%) had immediate complications following the procedure, none of which were life threatening. Minor bleeding was the most common complication. Among the study population, 42 (50.6%) of the patients were discharged.

High prevalence of neurological conditions (63.9%), particularly intracranial hemorrhage (20.5%) and traumatic brain injury (9.6%), was observed in the study population. This aligns with various international and national studies conducted in neurosurgical patients, which have highlighted the association between severe brain injury and prolonged mechanical ventilation, necessitating tracheostomy. These patients often require prolonged mechanical ventilator support due to impaired neurological function, reduced airway protective reflexes, and delayed recovery.^7-9^

The majority of PDT procedures in our study were performed using the Griggs' guidewire dilator forceps (GWDF) technique (92.7%), with only a minority of cases (7.3%) was done using Ciaglia's Blue Rhino technique. Similar trends have been reported in other studies from Nepal, where the Griggs' technique is the preferred method.^11,12^ This preference may be attributed to its availability and familiarity of intensivists in our ICU.

The procedural aspects of our study demonstrate a median time to tracheostomy of 16 days, which exceeds the early tracheostomy window of a ≤7 days recommended in traumatic brain injury (TBI) guidelines and CENTER-TBI study and a ≤5-day in the SETPOINT 1 and 2 trials done among stroke patients.^8,13,14,15^ Brook et al. observed reduced duration of mechanical ventilation and hospitalization costs with early tracheostomy done within 10 days and Rumbak et al. demonstrated significantly lower ICU stay and mortality with ultra-early intervention (<48 hours).^16,17^ While these international studies increasingly favor early tracheostomy done within first week of intubation, the delay to time to tracheostomy may be due to several factors encountered in our setting including limited operating room availability due to high demand for elective and emergency procedures. Additionally, PDT are not performed during off hours in our institute.^10^

The complication rates following PDT are comparable to studies as a safe bedside procedure in our setup. The observed complication rate of 14.5% aligns with international standards of 12-18% in comparable prospective studies suggesting acceptable safety.^18-21^ Minor bleeding was the most common complication (10.8%) which stopped on compression alone. These rates are within the range reported in literature from similar settings.^10,11,12,22^ This safety profile may be attributed to the use of pre-procedural ultrasound screening of neck to assess local anatomy and routine use of fiberoptic bronchoscopic guidance for real time visualization which have shown to lower procedural complications.^23-26^

This study has several limitations. Data collection was limited by the retrospective nature of the study, with 16 patients excluded due to incomplete medical records. The relatively small sample size of 83 patients from a single center in Nepal, may not represent all populations. Without prospective follow up, important details regarding long term complications, actual decannulation rates and post discharge functional status assessment was not possible. Future multicentred, prospective studies with long term follow up are required to validate and extend these findings.

## CONCLUSIONS

Percutaneous dilatational tracheostomy represents a safe bedside procedure for airway management in critically ill patients requiring prolonged mechanical ventilation. The procedure demonstrates acceptable complication rates and favorable outcomes when performed in a tertiary care setting.
